# What Nurses’ Work–Life Balance in a Clinical Environment Would Be

**DOI:** 10.3390/healthcare13040427

**Published:** 2025-02-16

**Authors:** Sharifa M Alasiry, Fauzia Naif Alfridi, Hibah Abdulrahim Bahri, Hanan HamdanAlshehri

**Affiliations:** 1 College of Nursing, Majmaah University, Al-Majmaah 11952, Saudi Arabia; s.alasiry@mu.edu.sa; 2 Saudi Arabia Ministry of Health, P.O. Box 6291, Riyadh 52367, Saudi Arabia; falfridi1406@gmail.com; 3 Department of Medical and Surgical Nursing, Collage of Nursing, Princess Nourah bint Abdulrahman University, P.O. Box 84428, Riyadh 11671, Saudi Arabia; hhalshehri@pnu.edu.sa

**Keywords:** nursing, work engagement, work quality of life, hospital

## Abstract

Background/Objectives: Nurses’ workplaces are critical to both the levels and types of care nurses can deliver as well as to employee retention. The quality of the workplace environment is a significant predictor of employment outcomes, such as improved care and a decrease in nurses’ desire to leave their work. Moreover, a favourable work environment can significantly improve organisational performance. A healthy nursing workplace is a safe, empowering, and fulfilling place of employment in which all healthcare personnel work tirelessly for patients’ optimal health and wellness. The aim of this study is to identify the association between workplace environment and work engagement among nurses in clinical settings. Methods: A descriptive cross-sectional correlational design was used. Convenience sampling was used to recruit 349 nurses from various hospitals in the central northern region of Saudi Arabia. A four-part electronic questionnaire eliciting information on participants’ sociodemographic characteristics, job characteristics, work-related quality of life (WRQoL), and work engagement was collected. All ethical guidelines for scientific research on human beings were strictly followed. Results: The participants had average levels of WRQoL. However, they had generally moderate to high levels of work engagement, which reflects their moderate to low intention to leave work. The findings demonstrated a statistically significant positive link between the WRQoL and work engagement of the participants. Conclusions: When the nurses’ WRQoL improved, their work engagement increased significantly. Improving WRQoL will almost definitely enhance nurses’ work engagement, which will reduce nurses’ intentions to leave their employment. It is advised that nurses enhance their WRQoL in order to increase their engagement in the workplace, have fewer thoughts about leaving, and make fewer plans to leave.

## 1. Introduction

Over the past four decades, the workplace environment has influenced nurses’ turnover intentions, which has had an impact on patient outcomes [1,2,3,4].

The workplace environment and work engagement of nurses in clinical settings are critical components that influence both healthcare outcomes and the well-being of nursing professionals. The World Health Organization (WHO) has initiated projects aimed at improving these aspects, recognizing that a supportive work environment is essential for enhancing job satisfaction and retention among nurses. The WHO’s strategic framework emphasises the need for policies that foster healthy workplaces, thereby addressing the pressing human resource challenges that the healthcare sector faces globally [5].

In parallel, organisations such as the International Council of Nurses (ICN) and the Royal College of Nursing (RCN) in Great Britain are actively engaged in initiatives that advocate for the rights and welfare of nurses. The ICN has developed guidelines and resources focused on creating optimal work environments, promoting nurse engagement, and ensuring quality patient care [5]. Similarly, the RCN has launched campaigns aimed at improving workplace conditions, addressing issues such as workload, mental health support, and professional development opportunities for nurses [5]. Research on the quality of life and work of nurses is thus not merely a professional concern; it is vital for studies aimed at preventing the human resource crisis highlighted by the WHO in its strategic documents for achieving the Sustainable Development Goals by 2030.

Healthy work environments (HWEs) are characterised by (a) a high level of trust between management and staff members, (b) a culture that encourages communication and cooperation, and (c) employees who feel physically and psychologically safe and well [6]. An HWE fosters effective interaction, which contributes to task fulfilment, work engagement, and intention to remain. It has been confirmed that an HWE improves nurse satisfaction and patient outcomes [7]. Factors considered in the work environment include worker security, task security, effective communication among workers, recognition of outstanding effort and efficiency, and workers being highly motivated and involved in the organisation’s decision-making processes [8]. Moreover, the nursing practice environment is based on whether the organisational aspect of the workplace supports or hinders nurses’ professional work [3]. There has been an increasing realisation that improving working conditions in medical facilities is crucial in fostering a sustainable workforce, providing the best patient care, engaging nurses at work, and minimising staff turnover [9,10]. When nurses’ working circumstances improve dramatically, their desire to resign is reduced, and their work engagement increases [11,12]. In addition, recognising factors that create a good work environment, such as a favourable atmosphere, can help to lower turnover targets and boost nurse engagement with the administration and decision making of their organisations [13]. These factors include independence, environmental protection, good physician–nurse relationships, and support from the organisation [14,15]. Thus, a recent study showed that the work environment has a significant impact on nurse turnover in hospital settings [16]. Moreover, newly graduated nurses have been leaving workplaces when environmental modifications are needed within the first six months of work [17,18]. Furthermore, turnover intention is connected to level of education, marital status, employment, experience, and income [19]. According to Park and Min [20], the precursors of turnover intention in medical facility settings include, but are not limited to, work attitude, workplace stress, market circumstances, role stressors, and colleague support. These interconnected characteristics have diverse influences on each other. Understanding the dynamics of employee well-being is essential for maintaining a sustainable workforce and ensuring high-quality patient care. Three key concepts—work-related quality of life (WRQoL), work engagement, and turnover intentions—play a significant role in shaping the experiences of nurses in their work environments. Work-related quality of life (WRQoL) refers to the overall satisfaction and well-being of employees in their work environment, encompassing job satisfaction, work–life balance, and workplace conditions [21]. Work engagement is a positive state characterised by vigour, dedication, and absorption in one’s work, which are crucial for delivering high-quality care [21]. Turnover intentions are the likelihood that an employee will leave their job, serving as a predictor of actual turnover behaviour. High turnover intentions among nurses can lead to staffing shortages and negatively impact patient care [22].

Addressing these interconnected concepts is vital for healthcare organisations seeking to improve nurse retention and enhance the overall quality of care. By fostering a positive work environment that enhances WRQoL and promotes work engagement, organisations can effectively reduce turnover intentions among nurses, leading to a more stable and satisfied workforce. Ultimately, prioritizing these factors will benefit both nursing professionals and the patients they serve, creating a healthier healthcare system.

Worldwide, the nursing profession has one of the highest turnover rates; therefore, in order for the profession to progress, more research studies on improving nurse retention should be conducted [23,24,25]. One study investigating the quality of the nursing working environment and nurse turnover in a single setting showed that the majority of nurses who expressed satisfaction also said they intended to leave and that there was little correlation between the quality of nurses’ working lives and turnover [26]. However, to the best of the authors’ knowledge, no study on the association between the workplace environment and nurse turnover has been undertaken in Saudi Arabia. The aim of this study is to identify the association between workplace environment and work engagement among nurses in clinical settings.

The study objectives were as follows:

To evaluate the work-related quality of life (WRQoL) and work engagement of nurses working in hospitals.

To determine whether there is a relationship between nurses’ WRQoL and turnover intentions (work engagement) among nurses working in hospitals.

## 2. Materials and Methods

A descriptive cross-sectional correlational research design was used in this study.

### 2.1. Sample and Settings

The current study was conducted at all tertiary governmental hospitals (n = 9) in the central northern region of Saudi Arabia. Convenience sampling is a non-probability sampling approach in which respondents are selected from a particular population. All nurses worked in various departments of Ministry of Health hospitals. The study’s sample included both genders and was composed of nurses with one year of experience or more. Information was obtained from those who were easiest to reach or contact. In total, about 3700 nurses work in Ministry of Health hospitals in the Qassim region. The necessary sample size was therefore estimated to be 349 respondents from different departments of the Ministry of Health hospitals in the studied region (Appendix A).

### 2.2. Instruments

A survey was carried out using a self-administered questionnaire consisting of two scales. The first scale was the WRQoL scale [27], which consists of 24 questions rated on a Likert scale that ranges from 1 (strongly disagree) to 5 (strongly agree). The second scale was the Work Engagement scale [28], which consists of 17 items rated on a Likert scale ranging from 1 (almost never) to 6 (always). Furthermore, information was gathered about the demographic and work characteristics of the respondents. The WRQoL [27] and Work Engagement scales are publicly available scales that were adopted from previous validated research studies [28], respectively (Appendix A). As English is formally used among nurses of different nationalities working in government hospitals in Saudi Arabia, the questionnaire was administered in its original language, that is, English.

### 2.3. Procedure

In total, (n = 349) respondents from different hospitals took part in this study. The authors used an online link containing the questionnaire and respondent consent form. The questionnaire was sent via email to the nursing departments in each hospital, which forwarded it to the targeted nurses working in each hospital. Answers to the questionnaire were collected over three months, and three reminder emails were sent to the respondents.

### 2.4. Statistical Analysis

Initially, descriptive statistics were used to analyse the distribution of the results of the WRQoL and Work Engagement scales. Statistical Package for Social Sciences (SPSS) Version 26 was used to analyse the mean, standard deviation, or number and frequency of the sociodemographic and job characteristics, WRQoL, and work engagement of the respondents. A *t*-test was performed to determine whether there was a connection between the respondents’ WRQoL and levels of work engagement. In addition, Cronbach’s alpha was computed to confirm the internal consistency and reliability of the *t*-test.

## 3. Results

**Figure 1:**
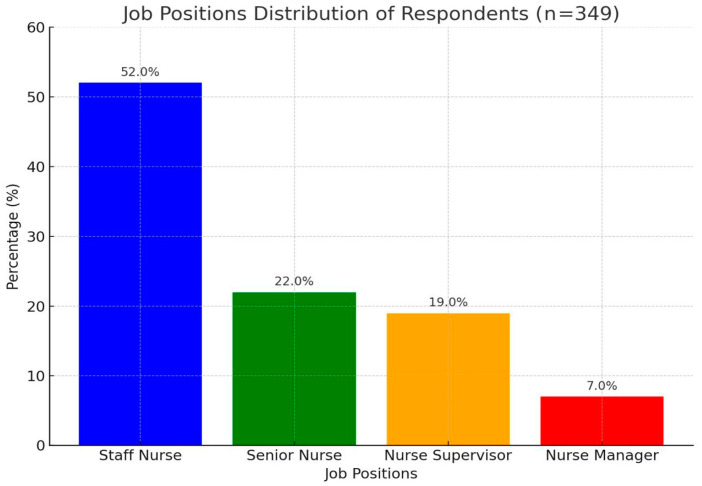
The job position distribution of the respondents (n = 349).

The basic distribution of the respondents’ characteristics is shown in Table 1. A total of (n = 349) nurses from various departments and hospitals participated in this study. The majority of the respondents were young people, with an average age of between 30 and 35 years (n = 130/37%), and almost two-thirds of the respondents (n = 221/63%) were female and married (n = 199/57.0%). Regarding their levels of education, more than half of the respondents (n = 182/52%) held a Bachelor’s degree in nursing science. The work characteristics of the respondents, including their work department, years of experience, and monthly income, are shown in Table 1. About one-fourth of the respondents were working in gynaecological wards (24%), and about 40% had 7 to 12 years of experience. Concerning monthly income, 43% of the respondents and about 52% of bedside staff nurses declared that their monthly income was more than 2500 USD per month, respectively, as shown in Figure 1.

### Reliability Analysis

The Cronbach’s alpha coefficient for the WRQoL was determined to be 0.97, and the same value was recorded for the Work Engagement Scale. This result demonstrates excellent internal consistency and reliability, indicating that the items within both domains consistently assess the same underlying constructs.

The statistical distribution of the respondents’ WRQoL is presented in Table 2. The mean score of each item was calculated. The highest mean score (3.17 ± 1.29) was for item number 1 (I have a clear set of goals and objectives that help me do my job) while the lowest mean score (2.81 ± 1.1) was for item number 22. (The conditions of work are good). In general, the respondents had average levels of WRQoL, as indicated by the mean score of the scale, which was found to be 2.932 ± 0.919 on a scale ranging from one to five.

The results regarding the respondents’ work engagement are presented in Table 3. The highest mean score of the items (3.66) was found for item number 10 (I am proud of the work that I do), while the lowest mean score (3.31) was found for item number 14 (I get carried away when working). Moreover, the respondents had generally moderate to high levels of work engagement, as indicated by the mean score of the scale (3.475 ± 1.211), which reflects that the respondents had a moderate to low intention to leave work.

Finally, for this study, we performed the Pearson correlation coefficient test to assess the correlation between WRQoL and work engagement. The result revealed a statistically significant positive correlation between the two variables (*p* = 0.000), meaning that when the mean of the total WRQoL Scale increased, the mean of the total Work Engagement Scale increased significantly, as shown in Table 4.

## 4. Discussion

The present study had two objectives: firstly, to evaluate the WRQoL and work engagement of hospital nurses; and secondly, to delve into the intricate relationship between WRQoL and turnover intentions (work engagement) among nurses working in hospitals. The results of the current survey indicate that WRQoL significantly affects nurses and their work engagement in their workplaces.

The study found that a significant number of the participants were young, with the majority being under 35 years old, which could reflect the age distribution of nurses in Saudi Arabian hospitals. This result is similar to a study conducted in Saudi Arabia exploring healthcare professionals’ opinions on the benefits and drawbacks of using social media in healthcare settings, which discovered that 45.7% of the nurses surveyed were between 31 and 40 years old [29].

Nursing in Saudi Arabia is traditionally considered a female-dominated profession, and this is reflected in the predominantly female gender of the nurses surveyed. These findings are supported by a previous study conducted in Saudi Arabia, which examined the relationship between work–life satisfaction and intention to quit among nurses in Saudi Arabia. Similarly, a study conducted in Saudi Arabia that explored the perspectives of nurse leaders on the challenges and limitations of using social media in healthcare settings found that females constituted 64% of the sample, further highlighting the predominance of women in the profession [29,30].

A significant number of participants in the present study had achieved high levels of education in nursing, with more than half holding a Bachelor’s degree. This finding indicates the high quality of the education provided to nurses in Saudi Arabia. These findings are consistent with another study that explored the perspectives of nurse leaders, which also found that most participants had a Bachelor’s degree specialising in nursing [29].

Regarding the participants’ WRQoL, the aspect with the highest mean score in the present study was the participants’ satisfaction with the presence of defined goals and targets for their work, which they considered crucial. However, the participants expressed the lowest levels of satisfaction with their overall working conditions in their workplace environment, which received the lowest positive responses in the survey. The findings of this study thus differ from a study conducted on nurses in Greece, which found that the highest mean score for WRQoL was related to the workplace [31]. Despite the differences, both the present study and the study done by Fradelos and collegues in 2020 concluded that nurses were generally unsatisfied with their working conditions [12].

A study that assessed the WRQoL of healthcare providers in eight hospitals in the northwest region of Italy used the WRQoL scale and found that healthcare employees who felt empowered to express their opinions and influence changes in their work area had the highest levels of job satisfaction [32]. This study’s findings suggest a positive association between employee empowerment and WRQoL in healthcare settings.

Furthermore, a study examining the WRQoL of general surgical residents in the United States contradicted the findings of a previous study. The study found that surgical residents were generally satisfied with and motivated by the career training provided by their organisation to perform their jobs safely and effectively. However, a survey conducted in the same study revealed dissatisfaction among the surgical residents with the resources and needs of their institutions [33]. This finding indicates a disconnect between the respondents’ satisfaction and the actual provision of resources. The study emphasises the contrasting results and highlights the dissatisfaction of surgical residents with the resources offered by their institutions.

In the current study, individuals reported a moderate degree of WRQoL and work engagement. This was evidenced by the mean score of 2.932 ± 0.919 out of 5 on the WRQoL scale, indicating moderate levels of satisfaction. A similar study conducted on nurses working in Greek hospitals supported these findings, with nurses reporting an average WRQoL of 3.37 ± 1.02, also reflecting a moderate level of satisfaction, albeit slightly higher than that found in the present study [31]. Overall, both studies indicate that participants reported a moderate degree of WRQoL and engagement.

The participants in the study reported a strong sense of pride in their nursing work but tended to get carried away while working. Most nurses had little intention to leave their jobs, indicating moderate to high levels of work engagement. A previous review study found that work engagement levels varied among nursing staff in different countries [34]. A recent study in Egypt found that the majority of staff nurses had low work engagement but that those with high work engagement experienced less psychological stress and were less likely to leave the profession [35].

This current study also showed a positive association between WRQoL and work engagement, as supported by research on nurse leaders in the United States that showed that engagement protects against burnout and stress while contributing to compassion satisfaction [36]. Meanwhile, a study from China found that nurses who underwent standardised training had a medium to high quality of work life and high work engagement. The same study found that burnout was negatively correlated with WRQoL and that professional identity and job engagement had positive correlations. There was also a positive association between WRQoL and work engagement, which was mediated by feelings of burnout and job identity [37].

A recent study in Saudi Arabian hospitals found that nurse managers exhibited high levels of organisational loyalty, quality of life outside of work, and job performance. However, staff nurses had lower levels of WRQoL, organisational loyalty, and job performance [38]. The current study’s results are consistent with the existence of a significant positive association between WRQoL and work engagement. Similar findings were observed in a study in Brazil, where healthcare professionals with high work engagement and satisfaction with residency programs were less likely to consider quitting [39]. In contrast, a study in Saudi Arabia showed a positive association between WRQoL and organisational commitment but found no significant relationships between WRQoL and work engagement [40]. Similarly, a study in India found a favourable association between WRQoL and work engagement among college professors, but it was not statistically significant [41]. The significant differences in study results across countries can be attributed to cultural attitudes, economic conditions, and variations in healthcare systems. Factors such as work professionalism, salary structures, job security, and differences in training and support systems influence nurses’ engagement levels and the quality of their work life [5]. Recognizing these contextual elements is vital for designing effective interventions to improve nurse well-being.

A notable limitation of this study arises from the use of nonprobability sampling, particularly through convenience samples, which poses a significant threat to the internal validity of the research design and negatively impacts the generalizability of the results. Conversely, employing random sampling methods would enhance the reliability of the study outcomes and help mitigate this limitation.

### Avenues for Future Research

Future studies could explore several avenues to build on these findings. For instance, longitudinal research could examine how WRQoL and work engagement evolve over time, particularly in response to changes in workplace policies or environmental factors. Additionally, qualitative studies might provide deeper insights into the specific challenges nurses face in their working conditions, as well as the factors that contribute to their sense of pride and engagement. Comparative studies across different regions or healthcare systems could enhance our understanding of cultural and contextual variables affecting WRQoL and work engagement.

## 5. Conclusions

In conclusion, the study investigated the work-related quality of life and work engagement levels of nurses in hospitals in the central northern region of Saudi Arabia. The findings showed that the highest mean score on the Work-Related Quality of Life Scale was for the item related to having clear goals and objectives at work, while the lowest mean score was the item for their satisfaction with their working conditions. The nurses had an average level of work-related quality of life and engagement. They revealed a job-related quality of life that was moderate, and they also exhibited a work engagement level that ranged from moderate to high. Furthermore, this research study found a significant positive correlation between work-related quality of life and work engagement levels. This suggests that when work-related quality of life increases, work engagement levels also increase significantly.

### Implication of Research Study

The current study advances theoretical discussions surrounding the relationship between WRQoL and turnover intentions in nursing. By establishing a positive association between these constructs, it contributes to the body of literature emphasizing the significance of work engagement as a protective factor against turnover. This aligns with existing theories on job satisfaction and retention, offering a nuanced perspective on how WRQoL influences nurses’ commitment to their roles.

The findings of this study also highlight the critical need for hospital administrations to prioritise enhancements in working conditions to improve the work-related quality of life (WRQoL) and work engagement among nurses. Given the reported dissatisfaction with overall working conditions, it is essential to implement targeted interventions that foster a supportive work environment, which may lead to increased job satisfaction and a reduction in turnover intentions. Additionally, the research emphasises the significance of employee empowerment, advocating for healthcare organisations to establish mechanisms that allow nurses to voice their opinions and influence workplace changes. This approach could further enhance their WRQoL and engagement levels. Additionally, this study emphasises the importance of creating a positive work environment that promotes work-related quality of life and work engagement among nurses. Thus, stakeholders in clinical settings should consider improving the work environment to increase job satisfaction and productivity and reduce nurse turnover. The findings advocate for systemic changes in clinical settings to support nursing staff, thereby benefiting both their well-being and patient outcomes. Overall, this study not only provides valuable empirical data to the field but also establishes a foundation for future research and practical interventions aimed at improving the professional lives of nurses in Saudi Arabia and beyond.

## Figures and Tables

**Table 1:** Demographic data of the respondents (n = 349).

Sociodemographic Data		n	%
Age	29 years or less	85	24.4%
	30–35 years	130	37.2%
	36–41 years	72	20.6%
	42–47 years	45	12.9%
	48 years or more	17	4.9%
Gender	Female	221	63.3%
	Male	128	36.7%
Social Status	Single	102	29.2%
	Married	199	57.0%
	Divorced	38	10.9%
	Widow	10	2.9%
Educational Level	Diploma degree	107	30.7%
	Bachelor’s degree	182	52.1%
	Master’s degree	51	14.6%
	Ph.D. degree	9	2.6%
Work Data		n	%
Workplace/department work	Paediatric ward	61	17.5%
	Gynaecology ward	84	24.1%
	Medical and surgical ward	78	22.3%
	Outpatient clinics	68	19.5%
	Others	58	16.6%
Years of experience	6 years or less	108	30.9%
	7–12 years	141	40.4%
	13–18 years	60	17.2%
	19 years or more	40	11.5%
Professional income	Less than 5000 SR	48	13.8%
	5000 to 10,000 SR	149	42.7%
	More than 10,000 SR	152	43.6%
	Less than 5000 SR	48	13.8%

**Table 2:** Work-Related Quality of Life (WRQoL) Scale.

Work-Related Quality of Life	Strongly Disagree		Disagree		Neutral		Agree		Strongly Agree		Mean	SD
	n	%	n	%	n	%	n	%	n	%		
1 I have clear goals and objectives that help me do my job.	28	8.0%	109	31.2%	60	17.2%	81	23.2%	71	20.3%	3.17	1.29
2. I feel like I can say what I think and make changes in my work area.	25	7.2%	98	28.1%	72	20.6%	132	37.8%	22	6.3%	3.08	1.09
3. I get to use my skills at work.	24	6.9%	105	30.1%	96	27.5%	86	24.6%	38	10.9%	3.03	1.12
4. I’m feeling good right now.	29	8.3%	94	26.9%	86	24.6%	115	33.0%	25	7.2%	3.04	1.10
5. My employer gives me the tools and freedom I need to balance work with my family life.	37	10.6%	96	27.5%	83	23.8%	95	27.2%	38	10.9%	3.00	1.19
6. My current work schedule and hours fit my personal needs.	52	14.9%	93	26.6%	87	24.9%	84	24.1%	33	9.5%	2.87	1.21
7. I often feel pressure at work.	48	13.8%	93	26.6%	80	22.9%	95	27.2%	33	9.5%	2.92	1.21
8. When I do a good job, my line manager tells me so.	47	13.5%	87	24.9%	96	27.5%	87	24.9%	32	9.2%	2.91	1.18
9. I’ve been feeling sad and unhappy lately.	55	15.8%	102	29.2%	77	22.1%	81	23.2%	34	9.7%	2.82	1.23
10. I’m happy with my life.	40	11.5%	87	24.9%	82	23.5%	103	29.5%	37	10.6%	3.03	1.20
11. I’m encouraged to learn new skills.	46	13.2%	105	30.1%	87	24.9%	83	23.8%	28	8.0%	2.83	1.17
12. I have a say in decisions that affect me at work.	41	11.7%	95	27.2%	96	27.5%	92	26.4%	25	7.2%	2.90	1.13
13. My boss gives me everything I need to do my job well.	38	10.9%	109	31.2%	88	25.2%	86	24.6%	28	8.0%	2.88	1.14
14. My boss actively promotes flexible work hours and schedules.	45	12.9%	95	27.2%	89	25.5%	96	27.5%	24	6.9%	2.88	1.15
15. My life is pretty good in most ways.	39	11.2%	99	28.4%	101	28.9%	85	24.4%	25	7.2%	2.88	1.12
16. I work in a safe place.	42	12.0%	89	25.5%	85	24.4%	101	28.9%	32	9.2%	2.98	1.18
17. Most of the time, things go well for me.	45	12.9%	87	24.9%	103	29.5%	84	24.1%	30	8.6%	2.91	1.16
18. I’m happy with the job opportunities here.	44	12.6%	92	26.4%	83	23.8%	97	27.8%	33	9.5%	2.95	1.19
19. I often feel too much stress at work.	47	13.5%	104	29.8%	87	24.9%	86	24.6%	25	7.2%	2.82	1.16
20. I’m happy with the training I get to do my current job.	37	10.6%	97	27.8%	93	26.6%	94	26.9%	28	8.0%	2.94	1.14
21. Lately, all things considered, I’ve been feeling pretty good.	38	10.9%	97	27.8%	100	28.7%	82	23.5%	32	9.2%	2.92	1.15
22. The conditions of work are good.	44	12.6%	101	28.9%	96	27.5%	92	26.4%	16	4.6%	2.81	1.10
23. I help make decisions that affect the public in my own community.	43	12.3%	91	26.1%	94	26.9%	98	28.1%	23	6.6%	2.91	1.14
24. I am happy with the overall quality of my work life.	50	14.3%	87	24.9%	90	25.8%	101	28.9%	21	6.0%	2.87	1.16
Total	984	12%	2312	28%	2111	25%	2236	27%	733	9%	2.932	0.919

**Table 3:** Work Engagement Scale for the respondents.

Work Engagement Scale	Almost Never		Rarely		Sometimes		Often		Very Often		Always		Mean	SD
	n	%	n	%	n	%	n	%	n	%	n	%		
1. I am overflowing with enthusiasm at work.	19	5.4%	117	33.5%	61	17.5%	50	14.3%	50	14.3%	52	14.9%	3.43	1.56
2. I find my work to be meaningful and purposeful.	11	3.2%	103	29.5%	61	17.5%	74	21.2%	76	21.8%	24	6.9%	3.50	1.37
3. Time flies when I’m working.	12	3.4%	85	24.4%	87	24.9%	85	24.4%	49	14.0%	31	8.9%	3.48	1.33
4. I feel powerful and vigorous at work.	15	4.3%	86	24.6%	85	24.4%	62	17.8%	73	20.9%	28	8.0%	3.50	1.38
5. I am excited about my job.	18	5.2%	84	24.1%	80	22.9%	77	22.1%	60	17.2%	30	8.6%	3.48	1.38
6. When I am working, I ignore everything else around me.	25	7.2%	74	21.2%	80	22.9%	75	21.5%	69	19.8%	26	7.4%	3.48	1.40
7. My job inspires me.	26	7.4%	77	22.1%	74	21.2%	72	20.6%	63	18.1%	37	10.6%	3.52	1.46
8. When I wake up in the morning, I want to go to work.	33	9.5%	82	23.5%	62	17.8%	67	19.2%	81	23.2%	24	6.9%	3.44	1.47
9. I am pleased when I am working intensely.	29	8.3%	79	22.6%	74	21.2%	90	25.8%	51	14.6%	26	7.4%	3.38	1.39
10. I am proud of the work that I do.	28	8.0%	67	19.2%	61	17.5%	73	20.9%	80	22.9%	40	11.5%	3.66	1.50
11. I am engrossed in my work.	28	8.0%	73	20.9%	76	21.8%	79	22.6%	62	17.8%	31	8.9%	3.48	1.43
12. I can work for extended amounts of time.	37	10.6%	70	20.1%	68	19.5%	66	18.9%	83	23.8%	25	7.2%	3.47	1.48
13. My job is tough to me.	39	11.2%	71	20.3%	64	18.3%	83	23.8%	53	15.2%	39	11.2%	3.45	1.52
14. I get carried away when working.	40	11.5%	88	25.2%	58	16.6%	76	21.8%	61	17.5%	26	7.4%	3.31	1.49
15. I am very resilient mentally at work.	30	8.6%	65	18.6%	72	20.6%	89	25.5%	60	17.2%	33	9.5%	3.52	1.43
16. It is difficult to remove myself from my job.	38	10.9%	67	19.2%	73	20.9%	72	20.6%	74	21.2%	25	7.2%	3.44	1.46
17. At work, I always continue, even when things do not go well.	22	6.3%	70	20.1%	76	21.8%	87	24.9%	63	18.1%	31	8.9%	3.55	1.39
Total	450	8%	1358	23%	1212	20%	1277	22%	1108	19%	528	9%	3.475	1.211

**Table 4:** Correlation between mean Work-Related Quality of Life Scale and Work Engagement Scale.

Correlation		Mean of Total Work-Related Quality of Life (WRQoL) Scale	Mean of Total Work Engagement Scale
Mean of total Work-Related Quality of Life (WRQoL) Scale	Pearson correlation	1	0.804 **
	Sig. (2-tailed)		0.000
	N	349	349
Mean of total Work Engagement Scale	Pearson correlation	0.804 **	1
	Sig. (2-tailed)	0.000	
	n	349	349

** Correlation is significant at the 0.01 level (2-tailed).

**Figure A1:**
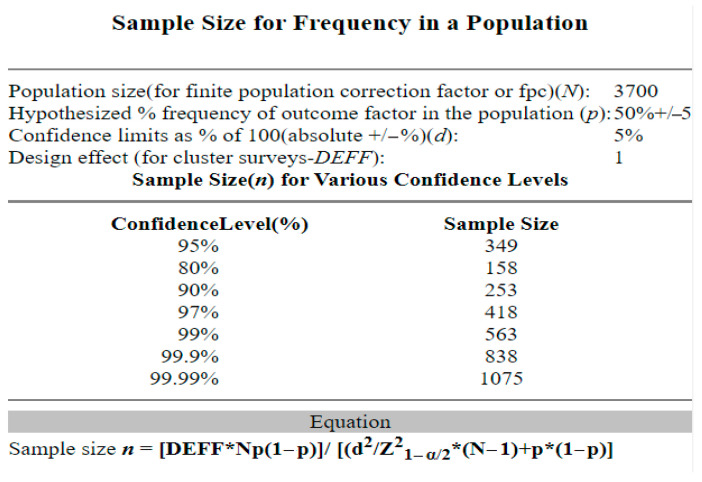
Sample size calculation.

## Data Availability

The data that support the findings of this study are available on request from the corresponding author. The data are not publicly available due to privacy or ethical restrictions.

## Appendix A. The Study Questionnaire

**Table A1:** Sociodemographic Characteristics.

Part 1: Sociodemographic Profile
Which category below includes your age?
24 > 29 years old
30 > 35 years old
36 > 41 years old
42 > 47 years old
48 or more
Gender
Female
Male
Social Status
Single
Married
Divorced
Widower
Your educational level
Diploma degree
Bachelor’s degree
Master’s degree
PhD

**Table A2:** Work Characteristics.

Part 2: Work Characteristics
**Which of the following is your department/workplace?**
Paediatric ward
Gynaecology ward
Medical and surgical ward
Outpatient clinic
Other
**Years of experience**
1 > 6 years
7 > 12 years
13 > 18 years
19 or more
**Salary:**
Less than 5000 SR
5000 to 10,000 SR
More than 10,000 SR
**Current position**
Staff nurse
Nurse supervisor
Head nurse
Nurse manager

**Table A3:** Scale 1: Work-Related Quality of Life (WRQOL).

Items	Strongly Disagree	Disagree	Neutral	Agree	Strongly Agree
1. I have a clear set of goals and aims to enable me to do my job					
2. I feel able to voice opinions and influence changes in my area of work					
3. I have the opportunity to use my abilities at work					
4. I feel well at the moment					
5. My employer provides adequate facilities and flexibility for me to fit work in around my family life					
6. My current working hours/patterns suit my personal circumstances					
7. I often feel under pressure at work					
8. When I have done a good job, it is acknowledged by my line manager					
9. Recently, I have been feeling unhappy and depressed					
10. I am satisfied with my life					
11. I am encouraged to develop new skills					
12. I am involved in decisions that affect me in my own area of work					
13. My employer provides me with what I need to do my job effectively					
14. My line manager actively promotes flexible working hours/patterns					
15. In most ways, my life is close to ideal					
16. I work in a safe environment					
17. Generally things work out well for me					
18. I am satisfied with the career opportunities available for me here					
19. I often feel excessive levels of stress at work					
20. I am satisfied with the training I receive in order to perform my present job					
21. Recently, I have been feeling reasonably happy, all things considered					
22. The working conditions are satisfactory					
23. I am involved in decisions that affect members of the public in my own					
24. I am satisfied with the overall quality of my working life					

**Table A4:** Scale 2: Work Engagement Scale. The following 17 statements are about how you feel at work (Work & Well-being Survey-UWES). Please read each statement carefully and decide if you ever feel this way about your job. If you have had this feeling, indicate how often you feel it by putting an X under the choice that best describes how frequently you feel that way.

Items	Almost Never	Rarely	Sometimes	Often	Very Often	Always
1. At my work, I feel bursting with energy						
2. I find the work that I do full of meaning and purpose						
3. Time flies when I’m working						
4. At my job, I feel strong and vigorous						
5. I am enthusiastic about my job						
6. When I am working, I forget everything else around me						
7. My job inspires me						
8. When I get up in the morning, I feel like going to work						
9. I feel happy when I am working intensely						
10. I am proud on the work that I do						
11. I am immersed in my work						
12. I can continue working for very long periods at a time						
13. To me, my job is challenging						
14. I get carried away when I’m working						
15. At my job, I am very resilient mentally						
16. It is difficult to detach myself from my job						
17. At my work, I always persevere, even when things do not go well						
